# Cathepsin D as a potential therapeutic target to enhance anticancer drug-induced apoptosis via RNF183-mediated destabilization of Bcl-xL in cancer cells

**DOI:** 10.1038/s41419-022-04581-7

**Published:** 2022-02-04

**Authors:** Seung Un Seo, Seon Min Woo, Seung-Soon Im, Younghoon Jang, Eugene Han, Sang Hyun Kim, Hongchan Lee, Hyun-Shik Lee, Ju-Ock Nam, Edward Gabrielson, Kyoung-jin Min, Taeg Kyu Kwon

**Affiliations:** 1 Department of Immunology, School of Medicine, Keimyung University, Daegu, 42601 South Korea; 2 Department of Physiology, School of Medicine, Keimyung University, Daegu, 42601 South Korea; 3 Department of Biology and Chemistry, Changwon National University, Changwon, 51140 South Korea; 4 Department of Internal Medicine, School of Medicine, Keimyung University, Daegu, 42601 South Korea; 5 Department of Pharmacology, School of Medicine, Kyungpook National University, Daegu, 41566 South Korea; 6 BK21 FOUR KNU Creative BioResearch Group, School of Life Sciences, Kyungpook National University, Daegu, 41566 South Korea; 7 Department of Food Science and Biotechnology, Kyungpook National University, Daegu, 41566 South Korea; 8 Department of Pathology, Johns Hopkins University School of Medicine and the Sidney Kimmel Comprehensive Cancer Center at Johns Hopkins, Baltimore, MD 21231 USA; 9 New Drug Development Center, Daegu-Gyeongbuk Medical Innovation Foundation (DGMIF), Daegu, 41061 South Korea; 10 Center for Forensic Pharmaceutical Science, Keimyung University, Daegu, 42601 South Korea

**Keywords:** Targeted therapies, Apoptosis

## Abstract

Cathepsin D (Cat D) is well known for its roles in metastasis, angiogenesis, proliferation, and carcinogenesis in cancer. Despite Cat D being a promising target in cancer cells, effects and underlying mechanism of its inhibition remain unclear. Here, we investigated the plausibility of using Cat D inhibition as an adjuvant or sensitizer for enhancing anticancer drug-induced apoptosis. Inhibition of Cat D markedly enhanced anticancer drug-induced apoptosis in human carcinoma cell lines and xenograft models. The inhibition destabilized Bcl-xL through upregulation of the expression of RNF183, an E3 ligase of Bcl-xL, via NF-κB activation. Furthermore, Cat D inhibition increased the proteasome activity, which is another important factor in the degradation of proteins. Cat D inhibition resulted in p62-dependent activation of Nrf2, which increased the expression of proteasome subunits (PSMA5 and PSMB5), and thereby, the proteasome activity. Overall, Cat D inhibition sensitized cancer cells to anticancer drugs through the destabilization of Bcl-xL. Furthermore, human renal clear carcinoma (RCC) tissues revealed a positive correlation between Cat D and Bcl-xL expression, whereas RNF183 and Bcl-xL expression indicated inverse correlation. Our results suggest that inhibition of Cat D is promising as an adjuvant or sensitizer for enhancing anticancer drug-induced apoptosis in cancer cells.

## Introduction

Combination therapy has several advantages over monotherapy in the treatment of cancers. Synergistic combination therapy maximizes the effects of anticancer drugs, such as induction of cell death and inhibition of tumor growth and metastatic ability, and reduces their side effects [1, 2]. Although newer anticancer strategies, such as immunotherapy, have been developed, chemotherapy remains the mainstay of cancer treatment. For example, cisplatin is a standard treatment for several cancers, such as ovarian, cervical, and head and neck cancers and lymphoma [3]; however, it has severe toxic side effects and increases cisplatin resistance in cancer cells [4, 5]. Therefore, to mitigate such effects, cisplatin is administered in combination with other anticancer drugs, such as doxorubicin, paclitaxel, and gemcitabine [3]. Sorafenib, an inhibitor of several receptor tyrosine kinases, is approved for the treatment of renal and liver cancers [6]. Although sorafenib monotherapy is used for solid cancers, its combination with other agents, such as antiangiogenic agents, mTOR inhibitors, and EGFR inhibitors, is more widely used [7]. Combination treatment reduces the development of drug resistance and enhances the anticancer effects [8]. Furthermore, substantial spatial and temporal heterogeneity of tumors necessitates combination therapy. Various combination therapies are currently being used for cancer treatment and several others are under preclinical and clinical investigations.

Cathepsin D (Cat D), a protein predominantly localized in lysosomes, performs multiple biological functions, such as degradation of intracellular and extracellular proteins, regulation of cell death, and activation of inflammatory cells [9–12]. Its role in cancer cells is worth studying, considering its high expression in several cancers [13–19]. Cat D increases cancer invasion, metastasis, and angiogenesis [20–22], and its overexpression increases the risk of recurrence and death in female patients with breast cancer [23]. In addition, Cat D is required for migration and invasion of gastric and breast cancer cells [18]. However, its effect on cell death is controversial and depends on stimulators and cell context. Cat D protects cancer cells from acetate- and oxidative stress-induced cell death [24, 25]. In contrast, it potentiates cell death in anticancer drug-treated cells [26–28]. The role of Cat D has been well-known in breast cancer. Pro-Cat D is released from triple-negative breast cancer, and increases proliferation of cancer cells [29]. Extracellular Cat D induces proteolysis of SPARC C-terminal extracellular Ca^2+^ binding domain, leading to stimulation of migration and invasion in triple-negative breast cancer [30]. Cat D-targeted antibody inhibits tumor growth in triple-negative breast cancer [17]. Recently, the role of intracellular Cat D, but not of its secreted form, has been identified. In PyMT cells, Cat D deficiency delays tumor cell proliferation through inhibition of mTORC1 signaling [31]. Although the role of Cat D in cancer has been extensively reported, its functions and molecular mechanisms in cancer cell death remain unclear.

Herein, we investigated whether inhibition of Cat D could enhance anticancer drug-mediated apoptosis, and identified the molecular mechanisms that sensitize cancer cells to induce apoptosis in the selected combination treatments.

## Materials and methods

### Cell culture and materials

All human cancer cell lines (Caki, ACHN, DU145, and HeLa) were obtained from the American Type Culture Collection (ATCC; Manassas, VA, USA). The mouse kidney cells (TCMK-1) were gifted by Dr. T. J. Lee (Yeungnam University, Korea) and normal human mesangial cells were purchased from Lonza (Basel, Switzerland). All cells were cultured in Dulbecco’s modified Eagle’s medium containing 10% fetal bovine serum (Welgene, Gyeongsan, Korea), 1% penicillin–streptomycin, and 100 μg/ml gentamycin (Thermo Fisher Scientific, Waltham, MA, USA). Details of the reagents, antibodies, siRNAs, and plasmids are provided in Supplementary Table 1.

### Cell viability analysis

Cell viability was measured using the WelCount^TM^ Cell Viability Assay Kit (Welgene, Daegu, Korea). Caki cells were treated with each drug, separately and in combination. After 24 h, XTT reagent and 1% PMS reagent were added to each well, and cells were incubated for 1 h at 37 °C, thereafter. Cell viability was measured with a multiwell plate reader using an excitation filter at 450 nm. To calculate GI50 (50% growth inhibition) value, Caki and Caki/Cat D KO cells were seeded in 96-well cell culture plates at a density of 5000 cells/well. After drug treatment, cell viability was determined using a colorimetric Cell Counting Kit-8, and values were calculated using the GraphPad Prism 9 software.

### Tumor spheroid preparation

Caki and Caki/Cat D KO cells were seeded in ultra-low attachment round-bottom 96-well plates (Corning) at a density of 4000 cells/well. The plates were centrifuged at 3000 × *g* for 10 min and then incubated for 72 h. The indicated concentrations of compounds were added to the wells. After treatment, cytotoxicity was detected by treating the cells with Sytox Green for 30 min at 37 °C. Image was acquired using a high content/high throughput-imaging device, Operetta CLS (PerkinElmer).

### Flow cytometry analysis

Harvested cells were resuspended in 100 µl phosphate-buffered saline (PBS) and fixed using 200 µl of 95% ethanol at 4 °C. Thereafter, cells were resuspended in 1.12% sodium citrate buffer (pH 8.4) containing 12.5 μg of RNase for 30 min at 37 °C, after which 50 μg/ml of propidium iodide solution was added. The percentage of apoptotic cell population was determined using BD Accuri^TM^ C6 flow cytometer (BD Biosciences, San Jose, CA, USA).

### Western blot analysis

Cells were collected and lysed in RIPA lysis buffer, and the lysates were centrifuged at 13,000 × *g* for 15 min at 4 °C. The supernatants were collected and boiled with 5× sample buffer for 5 min at 95 °C. Proteins were separated by SDS-PAGE and transferred onto nitrocellulose membranes (GE Healthcare Life Sciences, Pittsburgh, PA, USA). Protein bands were detected using enhanced chemiluminescence reagent kit (EMD Millipore, Darmstadt, Germany).

### Human cathepsin D plasmids construction

Total RNAs were extracted from hTERT-RPE1 cells and cDNAs were synthesized using PrimeScript™ 1st strand cDNA Synthesis Kit (Takara Bio Inc., Japan). A full-length wild type (WT) human cathepsin D (GenBank: NM_001909.5) was cloned into pCS107 vector having restriction sites for Cla1 and Not1. Mutant constructs of human cathepsin D c.289G>A; p.Asp97Asn and c.883G>A; p.Asp295Asn were generated by site-directed mutagenesis and primers were designed to have mutated nucleotides. WT human cathepsin D was used as a PCR template and amplified PCR products were digested using DpnI restriction enzyme to remove methylated WT template. WT hCTSD primer (Forward: GAT CAT CGA TGC CAC CAT GCA GCC CTC CAG CCT TCT G, Reverse: GAT TCG CGG CCG CCT ACT TGT CGT CAT CGT CTT TGT AGT CGA GGC GGG CAG CCT CGG CGA). D97N hCTSD primer (Forward: TCA CAG TCG TCT TCA ACA CGG GCT CCT CC, Reverse: GGA GGA GCC CGT GTT GAA GAC GAC TGT GA), D295N hCTSD primer (Forward: TGT GAG GCC ATT GTG AAC ACA GGC ACT TCC C, Reverse: GGG AGG TGC CTG TGT TCA CAA TGG CCT CAC A).

### Nuclear condensation and DNA fragmentation assay

Cancer cells were treated with Pep A in the presence or absence of TRAIL for 24 h. Nuclei were examined using 500 ng/ml 4′6′-diamidino-2-phenylindole reagent (Roche, Mannheim, Germany) under a fluorescence microscope (Carl Zeiss, Jena, Germany); fragmented DNA was detected using the cell death detection ELISA plus kit (Roche, Basel, Switzerland).

### Asp-Glu-Val-Asp-ase (DEVDase) activity assay

Cell lysates were incubated with a reaction buffer containing 5 μM of a caspase substrate (Asp-Glu-Val-Asp-chromophore-p-nitroanilide (DEVD-pNA)) in 96-well microtiter plates for 2 h at 37 °C. DEVDase activity was measured based on absorbance at 405 nm.

### Release of cytochrome *c*

For the analysis of cytochrome *c* release [32], cell lysates were centrifuged at 13,000 × *g* for 15 min at 4 °C. The supernatants (cytosolic extract) and the pellets (mitochondrial extract) were collected. The amount of cytochrome *c* released into the cytoplasm was analyzed using western blotting.

### Reverse transcription-polymerase chain reaction and quantitative PCR

Reverse transcription-polymerase chain reaction and real time-PCR were performed as described previously [33]. Sequences of the primers are detailed in Supplementary Table 1.

### Immunoprecipitation assay

Cells were collected, washed with PBS, lysed with RIPA lysis buffer containing 10 mM N-ethylmaleimide (EMD Millipore) and 1 mM PMSF, and then sonicated on ice for protein extraction. After sonication, cell lysates were centrifuged at 13,000 × *g* for 15 min at 4 °C. The supernatants were incubated with 1 μg of anti-Bcl-xL antibody overnight at 4 °C, and then attached to 20 μl of Protein G agarose bead by mixing on a rotator at 4 °C for 2 h. Cell lysates were washed with RIPA lysis buffer containing 10 mM N-ethylmaleimide and 1 mM PMSF, and boiled in 2× sample buffer for 10 min. Protein–protein interactions were checked by western blot analysis.

### Ubiquitination assay

The assay was performed as described previously [34]. Cells were cotransfected with HA-tagged ubiquitin (HA-Ub) and Myc-RNF183 plasmid, and treated with MG132 for 12 h. Immunoprecipitation was performed using anti-Bcl-xL, and ubiquitination of endogenous Bcl-xL was checked using HRP-conjugated anti-Ub antibody under denaturing conditions.

### Transfection and luciferase assay

Cells were transfected with siRNA using Lipofectamine RNAiMAX (Thermo Fisher Scientific), and transiently transfected with promoter plasmid using Lipofectamine^TM^ 2000 (Thermo Fisher Scientific). After treatment, the cells were collected and lysed in a lysis buffer (25 mM Tris-phosphate, pH 7.8, 2 mM EDTA, 10% glycerol, and Triton X-100). The supernatant was analyzed with dual-luciferase reporter reagent (Promega, Madison, WI, USA) according to the manufacturer’s recommendations.

### Generation of cathepsin D KO cells

Two CRISPR sgRNAs were designed using the CRISPR designing tool, their sequences being: oligomer1, 5′-CAC CGA TGG GCC CCT CGG TCA CGG C-3′ and oligomer2, 5′-AAA CGC CGT GAC CGA GGG GCC GAT C-3′. The cathepsin D KO cell lines were established by transfecting these sgRNAs into Caki cells using the Lipofactor-pMAX reagent; cells were selected on 0.5 μg/ml puromycin, and cathepsin D KO efficiency was checked using western blot analysis. We established three Cat D KO cell line, and mainly used Cat D KO #1 line.

### Chromatin immunoprecipitation assay

This assay was performed using the acetyl-histone H4 immunoprecipitation (ChIP) assay kit according to the manufacturer’s instructions (EMD Millipore). ChIP assays were performed using the two putative NF-κB-binding sequences in the RNF183 promoter region. Sequences of PCR primers were as follows: (sense) 5′-CCT CTT CAC ATC TAC AAA GAC GAA-3′ and (antisense) 5′-CTG CTT GGA GCC AAG GCC AGA GAG-3′. Products were detected on 2% agarose gel under UV light.

### Proteasome activity assay

The assay was performed as described previously [35]. Briefly, cells were collected, washed with PBS, and lysed. Thereafter, 1 μg of protein was incubated with Suc-LLVY-AMC (Biomol International, Plymouth Meeting, PA, USA) for 30 min at 37 °C. Chymotrypsin-like proteasome activity was measured using a fluorometric plate reader with excitation and emission filters at 380 and 440 nm, respectively.

### Nuclear and cytoplasmic extraction assay

After treatment, cells were washed with Tris-buffered saline, lysed in lysis buffer (10 mM HEPES at pH 7.9, 0.1 mM EDTA, 0.1 mM EGTA, 10 mM KCl, 1 mM DTT, and 0.5 mM PMSF), and 10% NP-40 was added to it. The lysate was centrifuged at 13,000 × *g* for 10 min at 4 °C to obtain the supernatants (cytosolic extracts) and pellets, which were incubated in a buffer (20 mM HEPES at pH 7.9, 1 mM EDTA, 0.4 M NaCl, 1 mM DTT, and 1 mM PMSF) with mixing using a rotator for 1 h at 4 °C. After centrifugation, the nuclear extract was obtained as the supernatant.

### Nuclear translocation assay

The treated cells were fixed with 4% paraformaldehyde on glass slides for 30 min at room temperature, washed with PBS, and permeabilized with 1% Triton X-100 for 1 min at room temperature. After washing with PBS, the slides were stained with anti-Nrf2 antibody overnight at 4 °C. The slides were subsequently incubated with an FITC-conjugated secondary antibody for 1 h, washed with PBS, and mounted on coverslips with ProLong™ Gold antifade mountant with DAPI (Molecular Probes, Eugene, OR, USA). The localization of Nrf2 was determined under LSM 510 multiphoton confocal microscope (Zeiss).

### ROS production

To measure intracellular ROS production, 2′,7′-dichlorodihydrofluorescein diacetate was used. Caki cells were treated with Pep A and incubated with 2′,7′-dichlorodihydrofluorescein diacetate fluorescent dye for 10 min at 37 °C. Green fluorescence was measured using a fluorescence microscope (Zeiss) and FACSCanto^TM^ flow cytometer (BD Biosciences).

### Patient specimens

A total of 40 patients diagnosed with RCC were included for retrospective study. Renal cell carcinoma tissues were collected from patients undergoing surgery at Keimyung University Dongsan Medical Center (Daegu, Korea), immediately frozen in liquid nitrogen, and stored at −196 °C until western blot analysis. Tissue samples were provided by the Biobank of Keimyung University Dongsan Hospital Biobank (IRB-2019-11-040).

### Animals

Male BALB/c-nude mice were purchased from the Central Lab Animal Inc. (Seoul, Korea). They were acclimatized for 1 week before the experiments and kept at 25 ± 2 °C under a relative humidity of 55 ± 5% and a 12 h light/dark cycle. The study protocol was approved by the IRB (KM-2015-03R2) Keimyung University ethics committee.

### In vivo xenograft model and TUNEL assay

Experiments were performed as described previously [36]. Mice were subcutaneously injected with Caki cells (5 × 10^6^) and assigned to the following experimental groups (6 mice per 1 group): vehicle, 3 mg/kg Pep A (20% DMSO + PBS), 3 mg/kg GST-TRAIL, and combination of Pep A and GST-TRAIL. The mice were administered intraperitoneal injection thrice a week for 16 days. Apoptosis was detected by TUNEL assay, using the ApopTag^TM^ fluorescein in situ apoptosis detection kit (EMD Millipore).

### Statistical analysis

Data were analyzed with one-way ANOVA and post hoc comparisons (Student–Newman–Keuls) using the Statistical Package for Social Sciences 22.0 software (SPSS Inc., Chicago, IL, USA).

## Results

### Cat D is highly expressed in renal tumor tissue

**Fig. 1:**
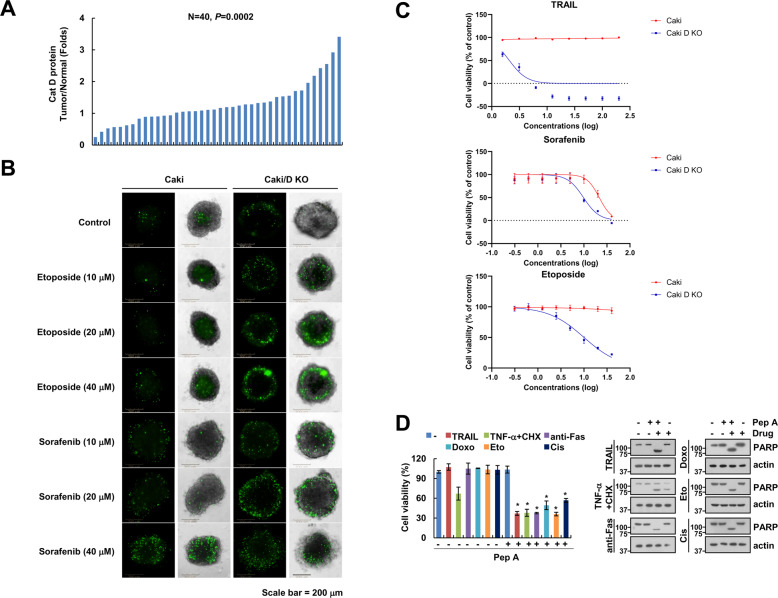
Cathepsin D (Cat D) is highly expressed in renal tumor tissue. **A** The levels of Cat D expression were detected by western blot analysis in 40 paired primary renal tumor tissues and corresponding normal adjacent ones. Data are shown as the fold change in renal cancer tissues (tumor) relative to adjacent normal tissues (normal). A value higher than 1 on the Y-axis indicates high expression in tumor tissue whereas a value lower than 1 shows low expression. (We analyzed the same protein samples in (**A**), Supplementary Fig. S1 and Fig. 9A). **B** Caki and Caki/Cat D knockout (KO) spheroids were treated with the indicated concentrations of etoposide and sorafenib for 24 h. Cell death was detected with Sytox green. **C** Caki and Caki/Cat D KO cells were treated with the indicated concentrations of TRAIL, sorafenib, and etoposide for 24 h. Growth inhibition was detected using a Cell Counting Kit-8 (*n* = 3). **D** Caki cells were treated with a combination of 50 ng/ml TRAIL, 2.5 ng/ml TNF-α plus 2.5 μg/ml cycloheximide (CHX), 750 ng/ml anti-Fas, 1 μM doxorubicin (Doxo), 3 μg/ml etoposide (Eto), and 30 μM cisplatin (Cis) in the presence or absence of 2 μM pepstatin A (Pep A) for 24 h. Cell viability was analyzed using an XTT assay kit. Values in the graphs (**D**) represent mean ± SD of three independent experiments (*n* = 3). *p < 0.01 compared to the control.

First, we assessed protein expression of Cat D was detected in 40 paired human renal carcinoma and adjacent normal kidney tissue samples. Data are shown as the fold change in renal cancer tissues (tumor) relative to adjacent normal tissues (normal). The Cat D levels were significantly higher in tumor tissues (*p* < 0.0214); 67.5% (27/40) of the carcinoma samples exhibited high levels of Cat D as compared to paired normal tissues (Fig. 1A and Supplementary Fig. S1). Next, we investigated if targeting Cat D would increase the anticancer effect of combination treatment with anticancer drugs. In tumor spheroids, Cat D knock out (KO) enhanced etoposide- and sorafenib-induced cell death (Fig. 1B). To validate these findings, Caki cells were treated with anticancer drugs in 2D culture. TRAIL, etoposide, and sorafenib dosing resulted in a GI50 value >20 μM whereas the GI50 values in Caki/Cat D KO cells were decreased (9.78 ± 1.7, 10.01 ± 1.34, and 2.081 ± 1.101 μM for sorafenib, etoposide, and TRAIL, respectively) (Fig. 1C). Moreover, Cat D inhibition by pepstatin A (Pep A) sensitized cancer cells to cell death induced by sublethal dosages of several anticancer drugs (Fig. 1D). Because a proper combination increases the effectiveness of the anticancer drugs used in a combination, the dosage of individual drugs, and thereby, their side effects, can be reduced. These results suggested that Cat D may be a useful adjuvant for inducing cancer cell death.

### Inhibition of Cat D enhances anticancer drug-induced apoptosis in cancer cells

**Fig. 2:**
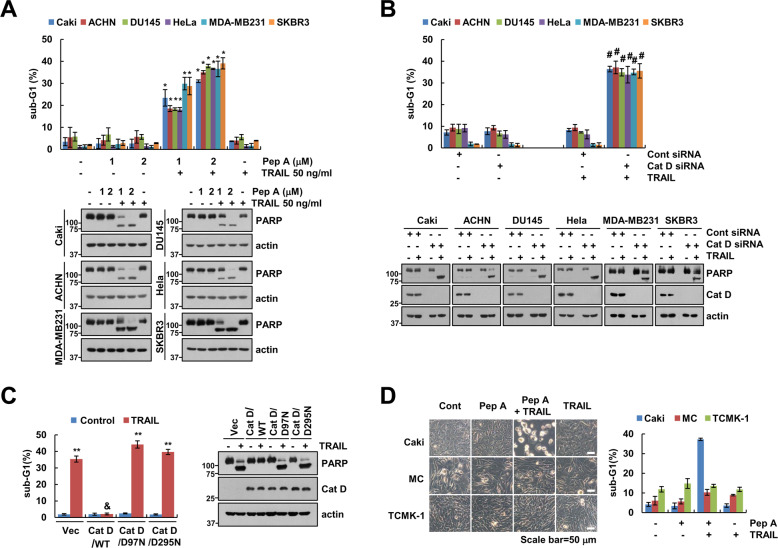
Inhibition of Cat D sensitizes cancer cells to anticancer drugs. **A** The indicated cancer cell lines were treated with 50 ng/ml TRAIL in the presence or absence of the indicated concentrations of pepstatin A (Pep A) for 24 h. **B** The indicated cancer cell lines were transfected with control (Cont) or Cat D siRNA and treated with 50 ng/ml TRAIL for 24 h. **C** Caki/Cat KO cells were transfected with vector, Cat D WT, or Cat D mutants (D97N and D295N), and treated with 50 ng/ml TRAIL for 24 h. **D** Caki, mesangial (MC), and TCMK-1 cells were treated with 50 ng/ml TRAIL in the presence or absence of 2 μM Pep A for 24 h. Cell morphology was assessed using a light microscope. Apoptosis was determined by flow cytometric analysis of sub-G1 populations (**A**–**D**) and PARP cleavage was determined by Western blot (**A**–**C**). *p < 0.01 compared to the control. **p < 0.01 com*p*ared to the control/Vec. ^&^*p* < 0.01 compared to the TRAIL-treated/Vec. ^#^*p* < 0.01 compared to TRAIL in Cont siRNA.

To investigate whether the increase in anticancer drug sensitivity upon Cat D inhibition is a common phenomenon in cancer cells, we conducted experiments using TRAIL. Human renal (Caki and ACHN), prostate (DU145), cervical (HeLa), and breast (MDA-MB231 and SKBR3) carcinoma cells showed dose-dependent increase in apoptosis upon treatment with TRAIL and pepstatin A (Pep A; a Cat D inhibitor) (Fig. 2A). Knockdown (KD) of Cat D also dramatically induced TRAIL-mediated apoptosis in cancer cells (Fig. 2B). To further confirm the effect of Cat D on TRAIL-induced cell death, we performed a reconstitution experiment by wild-type and a catalytically inactive mutant (D97N and D295N) of Cat D in Cat D KO cells. Ectopic expression of WT Cat D completely inhibited TRAIL-induced cell death in Cat D KO cells. However, mutants (D97N and D295N) did not inhibit TRAIL-induced apoptosis in Cat D KO cells (Fig. 2C). Furthermore, treatment of normal cells with sublethal dosages of TRAIL in the presence of Pep A did not induce apoptosis and morphological changes (Fig. 2C). These results suggested that Cat D sensitizes multiple cancer cell types to anticancer drug.

### Bcl-xL destabilization is involved in the synergistic effect of Cat D inhibition and TRAIL

**Fig. 3:**
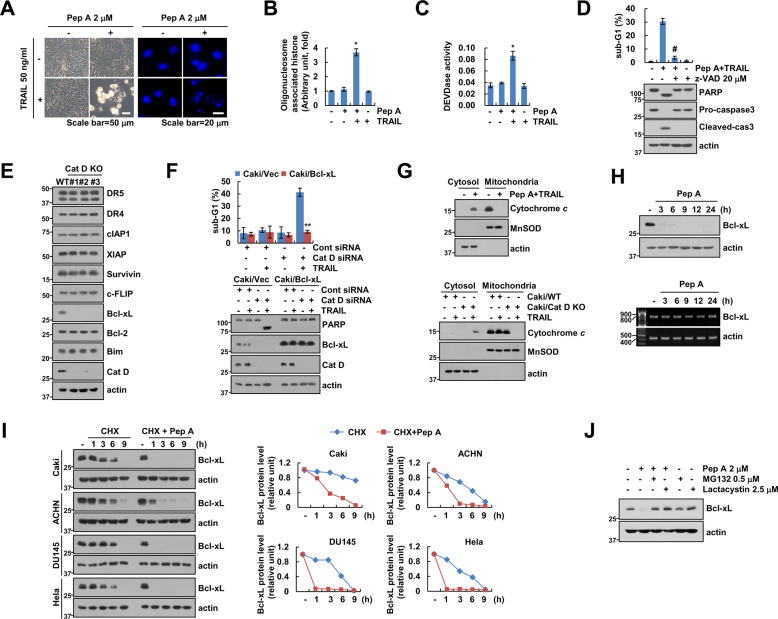
Inhibition of Cat D sensitizes cancer cells to TRAIL-mediated apoptosis through a decrease in Bcl-xL stabilization. **A**–**C** Caki cells were treated with 50 ng/mL TRAIL in the presence or absence of 2 μM Pep A for 24 h. Cell morphology and nuclear condensation were assessed under a microscope (**A**). Quantification of DNA fragments was performed using a DNA fragmentation assay kit (**B**). Caspase activity was measured using a DEVDase colorimetric assay kit (**C**). **D** Caki cells were treated with 2 μM Pep A and 50 ng/ml TRAIL in the presence or absence of a pan-caspase inhibitor, z-VAD-fmk (20 μM), for 24 h. **E** Expression of apoptosis-related proteins were determined in Caki/Cat D WT and KO cell lines. **F** Caki cells were transfected with pEBB (Caki/Vec) or pEBB-Bcl-xL (Caki/Bcl-xL) and Cat D siRNA, and then treated with 50 ng/ml TRAIL for 24 h. **G** Caki cells were treated with 2 μM Pep A and 50 ng/ml TRAIL for 24 h (upper panel). Caki and Caki/Cat D cells were treated with 50 ng/ml TRAIL for 24 h (lower panel). Cytochrome *c* release was analyzed by cytoplasmic fraction. MnSOD and actin were used as a mitochondrial and cytosol fraction marker, respectively. **H** Caki cells were treated with 2 μM Pep A for the indicated time periods. **I** Indicated cancer cells were treated with 20 μg/ml CHX, in the presence or absence of 2 μM Pep A, for the indicated time periods. Band intensity of Bcl-xL was analyzed using the ImageJ software. **J** Caki cells were pretreated with 0.5 μM MG132 and 2.5 μM lactacystin for 30 min, and treated with 2 μM Pep A for 24 h. Apoptosis was determined by flow cytometric analysis of sub-G1 populations and PARP cleavage assay (**D**, **F**). The protein and mRNA expression were measured by western blot analysis (**D**–**J**) and RT-PCR (**H**), respectively. Values in the graphs (**B**–**D**, **F**, **I**) represent the mean ± SD of three independent experiments (*n* = 3). *p < 0.01 compared to the control. ^#^*p* < 0.01 compared to the combinations of Pep A and TRAIL. **p < 0.01 compared to the TRAIL-treated Cat D siRNA in Caki/Vec cells.

Combined treatment with Pep A and TRAIL induced morphological changes and chromatin damage in nuclei (Fig. 3A) as well as DNA fragmentation (Fig. 3B). The treatment increased the activity of caspase 3, and a pan-caspase inhibitor reduced both cell death and cleavage of PARP and caspase 3 (Fig. 3C, D). Thus, the combined treatment induced apoptosis in cancer cells.

Balance between proapoptotic and antiapoptotic proteins plays critical roles in the induction of apoptosis. Cat D KO remarkably reduced the expression of Bcl-xL, although levels of other apoptosis-related proteins (DR4/5, cIAP1, XIAP, survivin, c-FLIP, Bcl-2, and Bim) were not altered in Caki cells. Pep A also reduced the expression of Bcl-xL in renal (Caki and ACHN), prostate (DU145), and cervical (HeLa) carcinoma cells (Fig. 3E and Supplementary Fig. S2A). We, therefore, investigated the significance of Bcl-xL. Ectopic expression of Bcl-xL remarkably blocked Cat D KD- or Pep A plus TRAIL-induced apoptosis (Fig. 3F and Supplementary Fig. S2B). Next, we investigated whether Pep A increases cytochrome *c* release in TRAIL-treated cells. Pep A plus TRAIL markedly increased cytochrome *c* release, and Cat D KO/KD also induced release of cytochrome *c* in TRAIL-treated cells (Fig. 3G and Supplementary Fig. S2C). Thus, inhibition of Cat D has a potentially critical role in enhancing chemosensitivity through downregulation of Bcl-xL.

To elucidate the mechanisms underlying the inhibition of Bcl-xL expression by Cat D inhibition, we detected its expression at mRNA and protein levels in Pep A-treated cells. Although Pep A decreased the expression of Bcl-xL within 3 h, its mRNA levels remained unaltered (Fig. 3H). When de-novo protein synthesis was blocked by cycloheximide (CHX), the expression of Bcl-xL was maintained until 6 h (Fig. 3I). However, combined treatment with Pep A and CHX induced degradation of Bcl-xL within 1 h in multiple cancer cell lines (Fig. 3I). Furthermore, proteasome inhibitors (MG132 and lactacystin) reversed the Pep A-induced downregulation of Bcl-xL (Fig. 3J). These data collectively suggested increased destabilization of Bcl-xL by Cat D inhibition in a proteasome-dependent manner.

### RNF183 is a key E3 ligase targeting Bcl-xL

**Fig. 4:**
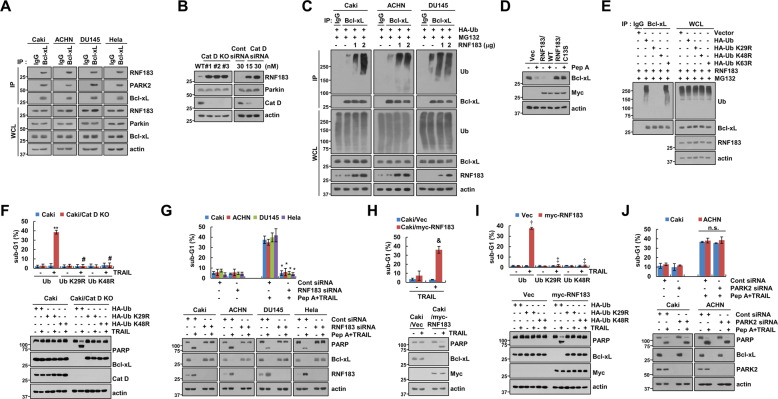
Cat D inhibition decreases the stability of Bcl-xL through upregulation of RNF183 expression. **A** Protein–protein interactions were checked using the immunoprecipitation assay with Bcl-xL antibody in various cancer cell lines. **B** Examination of protein expression in Caki and Cat D KO Caki cells (left panel). Caki cells were transfected with Cont or Cat D siRNA (right panel) for 24 h. **C** To analyze the ubiquitination of endogenous Bcl-xL, cells were cotransfected with Vec or myc-RNF183 and HA-ubiquitin (HA-Ub), and treated with 0.5 μM MG132 for 12 h. Immunoprecipitation was performed using an anti-Bcl-xL antibody under denaturing conditions. **D** Caki cells were transfected with pCMV3-ORF-Myc (Vec), pCMV3-ORF-Myc-RNF183/WT, and pCMV3-ORF-Myc-RNF183/C13S for 24 h, and treated with 2 μM Pep A for 24 h. **E** To analyze the ubiquitination of endogenous Bcl-xL, cells were cotransfected with Vec, HA-Ub, or HA-Ub mutants (K29R, K48R, and K63R) and myc-RNF183, and treated with 0.5 μM MG132 for 12 h. Immunoprecipitation was performed using an anti-Bcl-xL antibody. **F** Caki and Cat D KO Caki cells were transfected with HA-Ub and HA-Ub mutants (K29R and K48R), and treated with 50 ng/ml TRAIL for 24 h. **G** The indicated cancer cell lines were transfected with Cont or RNF183 siRNA, and treated with 2 μM Pep A plus 50 ng/ml TRAIL for 24 h. **H** Caki cells were transfected with pCMV3-ORF-Myc (Vec) or pCMV3-ORF-Myc-RNF183, and treated with 50 ng/ml TRAIL for 24 h. **I** Caki cells were cotransfected with Vec or myc-RNF183 with HA-Ub and HA-Ub mutants (K29R and K48R), and treated with 50 ng/ml TRAIL for 24 h. **J** The indicated cancer cell lines were transfected with Cont or PARK2 siRNA, and treated with 2 μM Pep A plus 50 ng/ml TRAIL for 24 h. Apoptosis was determined by flow cytometric analysis of sub-G1 populations and PARP cleavage assay (**F**–**J**). The protein expression was measured by western blot analysis (**A**–**J**). Values in the graphs (**F–J**) represent the mean ± SD of three independent experiments (*n* = 3). **p < 0.01 compared to the control in Ub-transfected Caki/Cat D KO. ^#^*p* < 0.01 com*p*ared to the TRAIL in Ub-transfected Caki/Cat D KO. *p < 0.01 compared to Pep A plus TRAIL in Cont siRNA. ^&^*p* < 0.01 compared to TRAIL in Caki/Vec. ^†^*p* < 0.01 compared to the control in Ub-transfected Caki/RNF183. ^‡^*p* < 0.01 compared to the TRAIL in Ub-transfected Caki^/^RNF183. n.s. = no significance.

We investigated the role of E3 ligases, RNF183 and Parkin [37, 38], in the destabilization of Bcl-xL upon Cat D inhibition. Although both these ligases bind to Bcl-xL (Fig. 4A), Cat D KO/KD only enhanced the expression of RNF183 (Fig. 4B). Therefore, we focused on the role of RNF183 in modulating Bcl-xL expression. RNF183 increased the ubiquitination of Bcl-xL in a dose-dependent manner in multiple cell lines (Fig. 4C). Ectopic expression of RNF183 dramatically induced the degradation of Bcl-xL, although a mutant form of RNF183 (RNF183/C13S) blocked Pep A-induced downregulation of Bcl-xL indicating that the catalytic activity of RNF183 is necessary for the degradation of Bcl-xL upon Cat D inhibition (Fig. 4D). Yanfang Wu et al. reported that polyubiquitination of Bcl-xL by RNF183 was blocked by K29R and K48R of Ub, but not K63R [37]. To investigate the type of Bcl-xL polyubiquitination by RNF183 in our system, we used mutant form of Ub lysine residues (K29R, K48R, and K63R). RNF183-mediated Bcl-xL polyubiquitination was inhibited by Ub-K29R and -K48R, but not K63R (Fig. 4E). In addition, Cat D KO increased TRAIL-induced apoptosis in Ub-transfected cells, but ectopic expression of Ub-K29R and -K48R completely blocked Bcl-xL degradation and TRAIL-induced apoptosis (Fig. 4F). To further validate the importance of RNF183, we investigated whether RNF183 could regulate Pep A plus TRAIL-induced cell death. KD of RNF183 inhibited Pep A plus TRAIL-induced apoptosis via inhibition of Bcl-xL degradation (Fig. 4G). Furthermore, ectopic expression of RNF183 induced the degradation of Bcl-xL, resulting in the induction of apoptosis by TRAIL alone (Fig. 4H). However, these effects were completely blocked by ectopic expression of Ub-K29R and -K48R (Fig. 4I). KD of PARK2 had no effect on cell death and Bcl-xL expression in Pep A plus TRAIL-treated cells (Fig. 4J). Our data, therefore, suggested that the induction of RNF183 by Pep A is critical in sensitization of cancer cells to anticancer drugs through the degradation of Bcl-xL.

### Activation of NF-κB enhances Cat D inhibition-mediated RNF183 expression

**Fig. 5:**
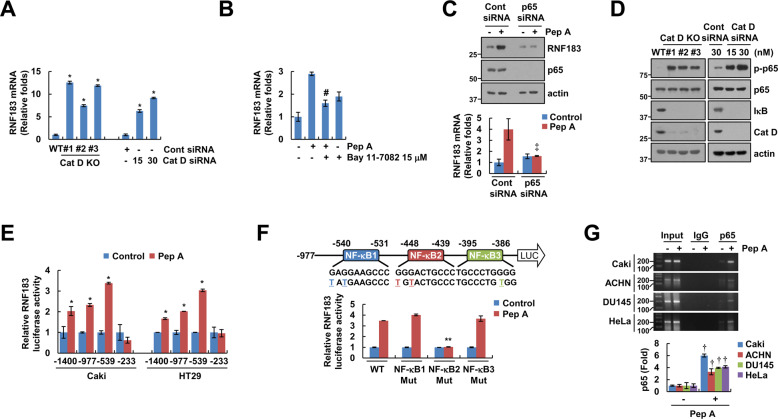
NF-κB is involved in Pep A-induced upregulation of RNF183. **A** Examination of RNF183 mRNA expression in Caki and Cat D KO/KD Caki cells. RNF183 mRNA expression was normalized using levels of actin. **B** Caki cells were pretreated with 15 μM Bay 11-7082 for 30 min, and then treated with 2 μM Pep A for 24 h. **C** Caki cells were transfected with Cont or p65 siRNA, and treated with 2 μM Pep A for 24 h. **D** Examination of protein expression was determined by Western blot in Caki and Cat D KO/KD Caki cells. **E** Caki and HT29 cells were transiently transfected with a plasmid harboring the luciferase gene under various lengths of RNF183 promoter, treated with 2 μM Pep A for 12 h, and analyzed for luciferase activity. **F** Caki cells were transiently transfected with a plasmid harboring the luciferase gene under the RNF183 WT or NF-κB mutant (Mut) promoter, treated with 2 μM Pep A for 12 h, and analyzed for luciferase activity. **G** The indicated cancer cell lines were treated with 2 μM Pep A for 12 h. Binding of RNF183 and NF-κB p65 was performed using a ChIP assay kit. The mRNA and protein levels were assessed by qPCR (**A**–**C**) and western blot analysis (**C**, **D**), respectively. Values in the graphs (**A**–**C**, **E**–**G**) represent the mean ± SD of three independent experiments (*n* = 3). *p < 0.01 compared to control. ^#^*p* < 0.01 com*p*ared to the Pep A. ^**‡**^*p* < 0.01 compared to the Pep A in Cont siRNA. **p < 0.01 compared to the Pep A in RNF183/WT promoter. ^†^*p* < 0.01 compared to the control in p65 IP.

To elucidate the mechanism of Cat D inhibition-mediated upregulation of RNF183, we examined the RNF183 mRNA levels. Cat D KO/KD markedly augmented the expression (Fig. 5A). Next, considering the presence of a putative NF-κB transcription factor-binding site in the RNF183 promoter, we checked the effect of an NF-κB inhibitor (Bay11-7082). Bay 11-7082 diminished the RNF183 mRNA expression in Pep A-treated cells (Fig. 5B). To further investigate the importance of NF-κB activation in RNF183 expression, cells were transfected with p65 siRNA. p65 KD remarkably blocked the upregulation of RNF183 expression by Pep A (Fig. 5C). Furthermore, Cat D KO/KD degraded IκB and enhanced p65 phosphorylation (Fig. 5D). Luciferase assay was performed using reporter plasmids containing various deletion mutants of the RNF183 promoter to identify the Cat D-responsive element. Pep A augmented the promoter activity of pRNF183/−1400, pRNF183/−977, and pRNF183/−539, but not of pRNF183/−233 (Fig. 5E). Because there are three putative NF-κB-binding sites from −540 to −1 in the promoter of RNF183, each NF-κB-binding site was mutated. When the second NF-κB-binding site (−448/−439) was mutated, Pep A did not induce the promoter activity of RNF183 (Fig. 5F). Therefore, the NF-κB-binding site activated by Pep A could be located at −448/−439 of the RNF183 promoter. To further confirm the involvement of NF-κB in RNF183 expression, the effect of Cat D inhibition on the direct binding of p65 to RNF183 promoter was examined using chromatin immunoprecipitation. Pep A remarkably enhanced the binding of p65 to the RNF183 promoter in multiple cancer cell lines (Fig. 5G), implying a critical role of NF-κB signaling in Cat D inhibition-induced expression of RNF183.

### Cat D inhibition induces the expression of proteasome subunits via Nrf2 transcriptional activity

**Fig. 6:**
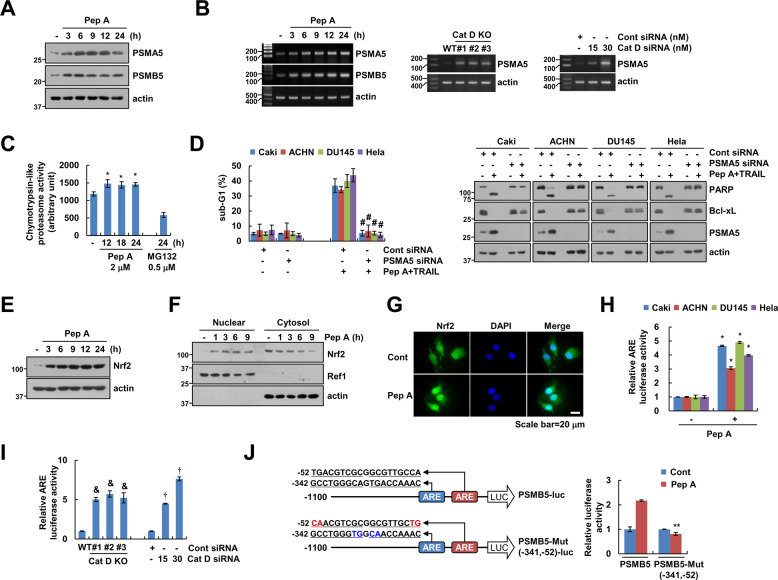
Cat D inhibition increases Nrf2-dependent upregulation of proteasome subunits. **A**–**C** Caki and/or Cat D KO/KD Caki cells were treated with 2 μM Pep A for the indicated time periods and analyzed for indicated protein and mRNA levels by western blot (**A**) and RT-PCR (**B**) analyses, respectively. Chymotrypsin-like proteasome activity was measured as described in the Materials and Methods section (**C**). **D** The indicated cancer cell lines were transfected with Cont or PSMA5 siRNA, and treated with 2 μM Pep A plus 50 ng/ml TRAIL for 24 h. **E**–**G** Caki cells were treated with 2 μM Pep A for the indicated time periods (**E**, **F**) or 6 h (**G**), and analyzed for the indicated proteins by western blot analysis (**E**). Nuclear and cytosolic fractions were prepared and measured by western blot analysis. Ref1 and actin were used as a nuclear and cytosol fraction marker, respectively (**F**). Fluorescence microscope was used to detect fluorescence intensity during Nrf2 translocation. Scale bar = 20 μm (**G**). **H** The indicated cancer cell lines were transiently transfected with a plasmid harboring the luciferase gene under the control of antioxidant response element (ARE) promoter. After transfection, the cells were treated with 2 μM Pep A for 12 h and analyzed for luciferase activity. **I** Caki and Cat D KO/KD Caki cells were transiently transfected with a plasmid harboring the luciferase gene under the control of ARE promoter and analyzed for luciferase activity. **J** Caki cells were transiently transfected with a plasmid harboring the luciferase gene under the control of PSMB5, or PSMB5-Mut (−341/−52) promoter and analyzed for luciferase activity. Apoptosis was determined by flow cytometric analysis of sub-G1 populations and PARP cleavage assay (**D**). The protein expression was measured by western blot analysis (**A**, **D**–**F**). Values in the graphs (**C**, **D**, **H**–**J**) represent the mean ± SD of three independent experiments (*n* = 3). *p < 0.01 compared to control. ^#^*p* < 0.01 com*p*ared to the combinations of Pep A and TRAIL in Cont siRNA. ^&^*p* < 0.01 compared to Caki/WT. ^†^*p* < 0.01 compared to Cont siRNA. **p < 0.01 compared to Pep A in PSMB5-Luc.

Cat D is mainly localized in lysosome, and plays a critical role in autophagy-lysosome degradation. However, RNF183-mediated Bcl-xL degradation is dependent on the ubiqutin-proteasome pathway. Therefore, we wondered whether inhibition of Cat D could modulate proteasome activity for degradation of ubiquitinated proteins. We investigate whether Cat D inhibition affects the expression of proteasome subunits and thereby modulate the proteasome activity [39–41]. Pep A, and Cat D KO/KD significantly increased the expression of the proteasome subunits, PSMA5 and PSMB5, at protein and/or mRNA levels (Fig. 6A, B). Chymotrypsin-like proteasome activity was also increased by Pep A treatment (Fig. 6C). siRNA-mediated KD of PSMA5 reversed the downregulation of Bcl-xL, and inhibited Pep A plus TRAIL-induced apoptosis in multiple cancer cell lines (Fig. 6D). We investigated the effect of Pep A on Nrf2 activation, a key transcription factor that induces the expression of proteasome subunits [42]. Pep A increased the expression (Fig. 6E) and nuclear translocation (Fig. 6F, G) of Nrf2. Furthermore, when we used an antioxidant response element (ARE) reporter vector, which contained a Nrf2-binding element, Pep A, and Cat D KO/KD remarkably increased ARE transcriptional activity (Fig. 6H, I). To confirm the involvement of ARE/Nrf2 signaling in the upregulation of proteasome subunits, we used luciferase reporter constructs with wild-type and mutant ARE, containing PSMB5 promoter. Although Pep A increased the luciferase activity of wild-type ARE containing PSMB5 promoter, that of mutant ARE containing PSMB5 promoter remained unaltered (Fig. 6J). Therefore, upregulation of proteasome subunit by Cat D inhibition could be involved in sensitization of cancer cells to anticancer drugs through the degradation of Bcl-xL.

### Activation of Nrf2 by Cat D inhibition is dependent on p62 expression

**Fig. 7:**
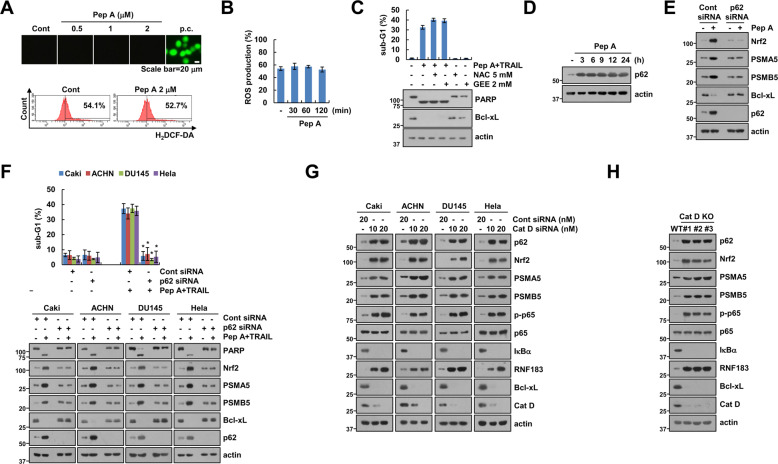
Upregulation of p62 by Cat D inhibition contributes to Nrf2 activation. **A**, **B** Caki cells were treated with 2 μM Pep A for 2 h (**A**) or for indicated times (**B**), and intracellular reactive oxygen species detected using fluorescence microscopy (**A**) and flow cytometry (**A, B**). **C** Caki cells were pretreated with 5 mM NAC or 2 mM GEE for 30 min, and then treated with 2 μM Pep A plus 50 ng/ml TRAIL for 24 h. **D** Caki cells were treated with 2 μM Pep A for the indicated times. **E**, **F** The indicated cancer cell lines were transfected with Cont or p62 siRNA and treated with 2 μM Pep A (**E**) or 2 μM Pep A plus 50 ng/ml TRAIL (**F**) for 24 h. **G** Examination of protein expression in Cont or Cat D siRNA-transfected cancer cell lines. **H** Examination of protein expression in Caki WT and three different Cat D KO cell lines. Apoptosis was determined by flow cytometric analysis of sub-G1 populations and PARP cleavage assay (**C**, **F)**. The protein expression was measured by western blot analysis (**C**–**H**). Values in the graphs (**B**, **C**, **F**) represent the mean ± SD of three independent experiments (*n* = 3). *p < 0.01 compared to the combinations of Pep A and TRAIL in Cont siRNA.

Reactive oxygen species (ROS) are the main activators of Nrf2. We, therefore, investigated whether Cat D inhibition activates Nrf2 via ROS production. Unexpectedly, Pep A did not increase ROS production (Fig. 7A, B). Furthermore, ROS scavengers (N-acetyl-L-cysteine and glutathione ethyl ester) did not inhibit Pep A plus TRAIL-induced apoptosis or downregulation of Bcl-xL (Fig. 7C). Nrf2 is noncanonically activated by p62 expression [43, 44]. The expression of p62 expression was dramatically increased by Pep A treatment (Fig. 7D). Moreover, KD of p62 reversed the upregulation of Nrf2 and proteasome subunits, resulting in the inhibition of Pep A plus TRAIL-induced apoptosis in multiple cancer cell types (Fig. 7E, F). We also found that upregulation of p62, Nrf2, PSMA5, PSMB5, and RNF183 proteins were observed in Cat D KD and KO cells, and these results were similar to those of Pep A treatment (Fig. 7G, H). Our data, collectively, indicated that Cat D inhibition induces Nrf2 activation via the upregulation of p62 expression.

### Combined treatment with Pep A and TRAIL reduces tumor growth and induces cell death in in vivo xenograft model

**Fig. 8:**
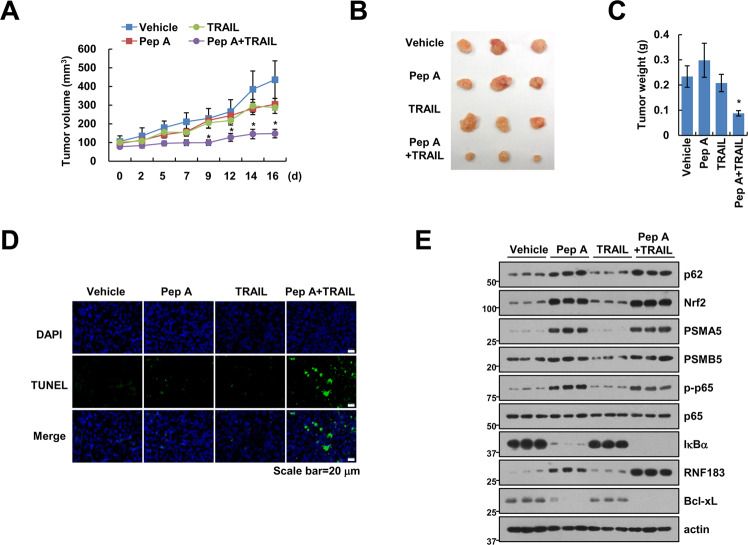
Combined treatment with Pep A and TRAIL reduces tumor growth in vivo. **A**–**E** Mice were treated with 5 mg/kg Pep A, 3 mg/kg GST-TRAIL, combinations of Pep A and GST-TRAIL, or vehicle for 16 days. The mice were randomly divided into 4 groups (*n* = 6). Tumor volume (**A**), size (**B**), and weight (**C**) were measured. TUNEL assays were performed to check apoptosis in vivo (**D**). Protein expression was measured by western blot analysis (**E**). *p < 0.01 compared to the vehicle.

To detect the synergistic effect of Cat D inhibition and TRAIL in an in vivo xenograft model, we subjected tumor-bearing mice to single (Pep A or TRAIL) or combined (Pep A plus TRAIL) treatments. Single treatment had no inhibitory effect on tumor size and weight, whereas combined treatment remarkably reduced the same (Fig. 8A–C). Furthermore, we detected TUNEL-positive cells in the combination treated-xenograft models (Fig. 8D). Using the samples collected from mice, we also detected the levels of protein related to the anticancer effects of Cat D inhibition. Pep A increased the expression of proteasome subunits and RNF183, and decreased that of Bcl-xL (Fig. 8E). Our data suggested that combination therapy using Cat D inhibitor and anticancer drug had synergistic effects in the in vivo model.

### Correlation of Cat D and RNF183/Bcl-xL in human RCC patient tissues

**Fig. 9:**
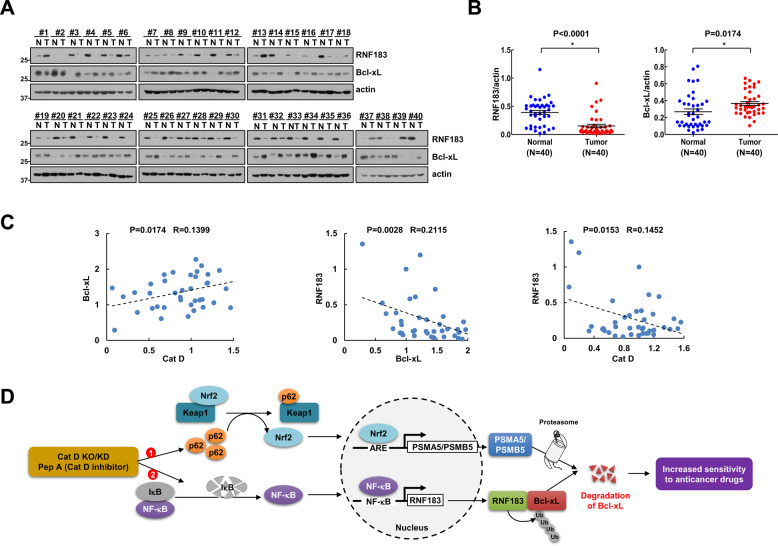
Analysis of protein levels and correlation of Cat D, Bcl-xL, and RNF183 in renal tumor tissue. **A**, **B** Investigation of the protein expression in 40 paired primary renal tumor tissues and corresponding normal adjacent ones (We analyzed the same protein samples in Fig. 1A and Supplementary Fig. S1). **C** Correlation analysis of protein expression of Cat D/Bcl-xL, RNF183/Bcl-xL, or RNF183/Bcl-xL. **D** Schematic representation of the mechanism of anticancer activity induced by Cat D inhibition.

Next, we examined protein expression of RNF183 and Bcl-xL in specimens of human renal clear carcinoma (RCC) tissues. The expression levels of RNF183 were downregulated in RCC tissues, while Bcl-xL expression was upregulated in RCC tumor tissues (Fig. 9A, B). When we quantified two proteins in all samples, 62.5% (26/40) of Bcl-xL was higher, whereas 77.5% (31/40) of RNF183 was lower in tumor tissues compared to adjacent normal tissues (Fig. 9B). Moreover, RCC tissues showed a positive correlation between Cat D and Bcl-xL expression, meanwhile, RNF183 revealed inverse correlation with Cat D and Bcl-xL (Fig. 9C).

## Discussion

We demonstrate that Cat D inhibition is a promising adjuvant or sensitizer for enhancing anticancer drug-induced apoptosis in cancer cells and present the underlying molecular mechanism. Inhibition of Cat D induced the downregulation of Bcl-xL expression. Two major mechanisms might be involved. First, Pep A might increase the expression of RNF183 through NF-κB activation. Second, Pep A could involve increasing the expression of proteasome subunits. Simultaneously, these two mechanisms could cause lowering of Bcl-xL expression upon Cat D inhibition, thereby enhancing anticancer drug-mediated cell death (Fig. 9D).

Recently, apoptosis-related proteins were reported to be post-translationally regulated. RNF183 and PARK2 are well-known Bcl-xL E3 ligases [37, 38]. Prolonged ER stress could increase the expression of RNF183 in an IRE1α-dependent manner [37]. Reduction of miR-7, a negative regulator of RNF183 expression, by IRE1α activation is related to the upregulation of RNF183 expression [37]. Contrarily, Gong et al. found that deletion of PARK2 frequently correlated with the amplification of Bcl-xL in human malignancies [38]. Investigations on whether E3 ligases are involved in reducing the expression of Bcl-xL by Cat D inhibition revealed that although both RNF183 and PARK2 bound to Bcl-xL under control conditions (Fig. 4A), the expression of RNF183 was significantly increased by Cat D KO/KD. Moreover, its KD blocked the downregulation of Bcl-xL in Pep A plus TRAIL-treated cells (Fig. 4B, G), suggesting a major role of RNF183 in the modulation of Bcl-xL expression by Cat D inhibition. The mechanism of regulation of RNF183 at the transcriptional level is not well understood. Maeoka et al. reported that nuclear factor of activated T cells 5 (NFAT5) induced the expression of RNF183 in inner-medullary collecting duct cells under hypertonic stress [45]. In our study, because the promoter of RNF183 had a putative NF-κB binding site, we tested whether NF-κB-induced the expression of RNF183. Cat D KO/KD induced p65 phosphorylation and IκB degradation (Fig. 5D). Furthermore, KD of p65 dramatically blocked the Pep A-induced expression of RNF183 (Fig. 5C). Therefore, NF-κB is a key transcriptional factor in Cat D inhibition-induced expression of RNF183. We identified NF-κB binding site, located at −448/−439 in the RNF183 promoter, to be critical for Pep A-induced upregulation of RNF183 levels (Fig. 5F). This could be a novel mechanism for regulating the expression of RNF183.

The role of Cat D in cell death appears to be the opposite, depending on its localization, condition of cells, and context in cancer cells and there are many possible reasons for this. Cat D mainly exists in lysosomes, but also localizes elsewhere, such as in the cytosol and extracellular space, under specific conditions. Therefore, the ability to modulate cell death may differ based on the localization of Cat D. For example, lysosomal membrane permeabilization (LMP) may be induced by stimuli, translocating Cat D from lysosome to cytosol, and cytosolic Cat D might be involved in inducing apoptosis [46–51]. In case of extracellular Cat D, it may nonproteolytically act as a mitogen, resulting in increased proliferation and migration of human omental microvascular endothelial cells and fibroblasts [52, 53]. It is also known as a survival regulator, related to the promotion of cancer growth in breast carcinoma [54]. Recently, Ketterer et al. reported the role of intracellular Cat D in tumor cells. Because Cat D KO mice are lethal, they used conditional KO PyMT mice and suggested that Cat D KO specifically in tumor cells induces dysregulation of mTORC1, resulting in the inhibition of proliferation [31]. Nuclear Cat D may act as a transcriptional repressor, a function not related to its catalytic activity. It increases the tricho-rhino-phalangeal syndrome 1 transcriptional repressor activity, resulting in the reduction of colony formation and cell cycle progression [55]. Therefore, functions of Cat D may differ depending on its localization. Moreover, differences in the cell context and conditions cannot be ruled out. To negate this possibility, we used multiple cell lines and diverse anticancer drugs. Anticancer drug-induced death was enhanced by Cat D inhibition in multiple cell lines (Figs. 1D and 2A). Therefore, the anticancer effect of Cat D inhibition was common under our experimental conditions. Finally, the concentrations of anticancer drugs used may induce different roles of Cat D. As mentioned above, the lethal dosage of anticancer drugs could induce persistent and strong LMP, resulting in cytosolic release of Cat D. However, sublethal or low dosage may have no or little effect on the induction of LMP. Furthermore, some drugs upregulate the expression of Cat D. Therefore, we cannot rule out the functional change, resulting from alteration in Cat D levels, balance between pro/pre and mature Cat D forms, or something novel that is not yet defined. Further investigations are required to clearly identify the role of Cat D in the regulation of cell death.

In conclusion, we clearly show that Cat D inhibition enhances anticancer drug-induced apoptosis through the degradation of Bcl-xL by upregulation of E3 ligase (RNF183) and proteasome subunit. Based on our findings, we suggest that strategies to control Cat D activity with anticancer drugs could promote cancer cell death.

## Supplementary information

SUPPLEMENTAL MATERIAL

Reproducibility checklist

co-authorship agreement

## Data Availability

The datasets used and analyzed during the current study are available from the corresponding author.
