# Supplementary material for: Cathepsin D as a potential therapeutic target to enhance anticancer drug-induced apoptosis via RNF183-mediated destabilization of Bcl-xL in cancer cells
Source: Cell Death Dis. 2022 Feb 4;13(2):115. doi: 10.1038/s41419-022-04581-7 (PMC8816936; doi:10.1038/s41419-022-04581-7)
Supplement: Supplementary file 1 — SUPPLEMENTAL MATERIAL [file 41419_2022_4581_MOESM1_ESM.docx]

**Supplemental material**

**Supplemental Figure S1**

**Supplemental Figure S2
Supplemental Table S1**

**
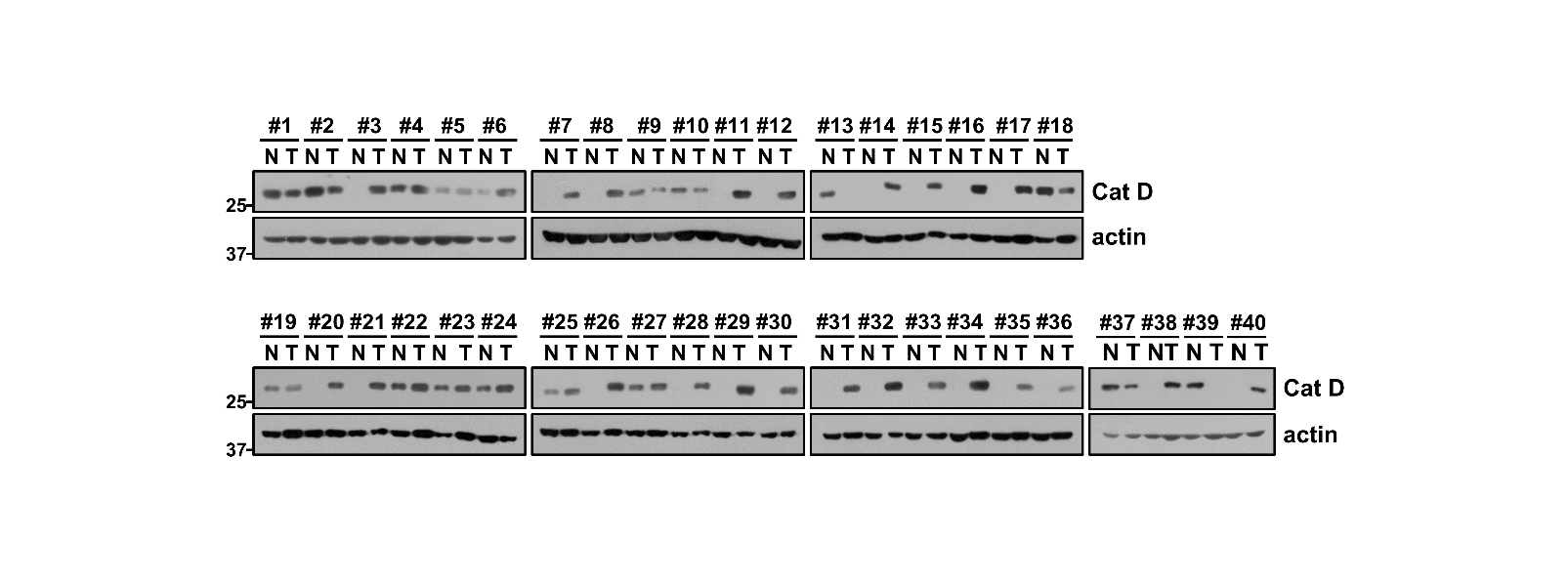
**

**Fig. S1** **Cat D protein expression was highly expressed in renal tumor tissues.** The expression levels of Cat D and actin were determined by Western blot in 40 paired primary renal tumor tissues and corresponding normal adjacent tissues.

**
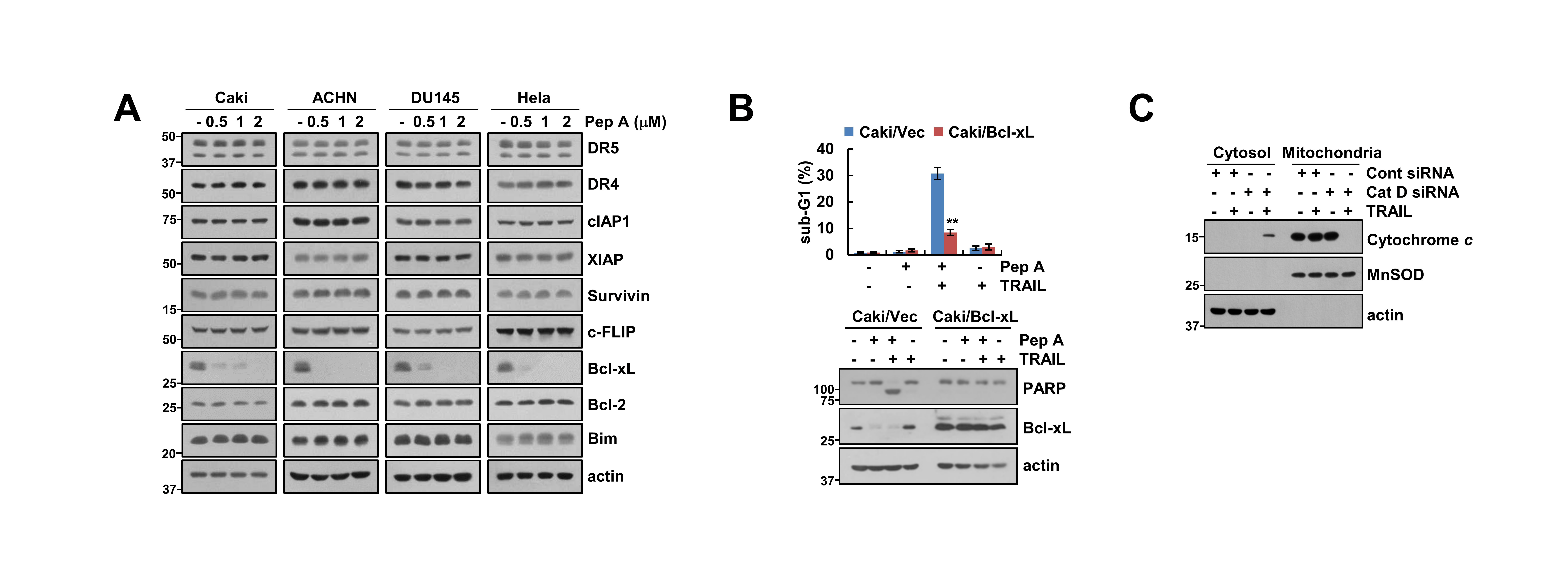
**

**Fig. S2** **Bcl-xL plays a critical role in sensitization cancer cells to anti-cancer drugs.** **A** The indicated cancer cell lines were treated with various concentrations of Pep A for 24 h. **B** Caki cells were transfected with pEBB (Caki/Vec) or pEBB-Bcl-xL (Caki/Bcl-xL) and treated with 50 ng/mL TRAIL in the presence or absence of 2 μM Pep A for 24 h. **C** Caki cells were transfected with Cont or Cat D siRNA and treated with 50 ng/ml TRAIL for 24 h. Cytochrome *c* release was analyzed by cytoplasmic fraction. MnSOD was used as a mitochondrial fraction marker. Apoptosis was determined by flow cytometric analysis of sub-G1 populations **(B)**. The protein expression was measured by western blot analysis (**A-C**). Values in the graph (**B**) represent the mean ± SD of three independent experiments (n=3). ^*^*p* < 0.01 compared to the Pep A plus TRAIL in Caki/Vec.

**Supplementary Table S1. Reagent and Antibody**

| Reagent or Antibody | Source | Identifier | |  |
| --- | --- | --- | --- | --- |
| Anti-Fas, clone CH11 | EDM Millipore | | Cat# 05-201 | |
| Anti-Fas, clone CH11 | EDM Millipore | | Cat# 05-201 | |
| b-actin | Sigma-Aldrich | | Cat# A2228 | |
| BAY 11-7082 | EDM Millipore | | Cat# 196870 | |
| Bcl-2 | Santa Cruz Biotechnology | | Cat# sc-7382 | |
| Bcl-xL | Cell Signaling Technology | | Cat# 2764 | |
| Bim | EMD Millipore | | Cat# AB17003 | |
| Cathepsin D | Santa Cruz Biotechnology | | Cat# sc-6486 | |
| c-FLIP | Enzo Life Sciences | | Cat# ALX-804-961-0100 | |
| cIAP1 | Cell Signaling Technology | | Cat# 4952 | |
| Cisplatin | Sigma-Aldrich | | Cat# P4394 | |
| Cleaved-caspase3 | Cell Signaling Technology | | Cat# 9661 | |
| Cycloheximide (CHX) | Sigma-Aldrich | | Cat# 01810 | |
| Doxorubicin | Sigma-Aldrich | | Cat# D1515 | |
| DR4 | Abcam | | Cat# ab8414 | |
| DR5 | Cell Signaling Technology | | Cat# 8074 | |
| Etoposide | Sigma-Aldrich | | Cat# E1383 | |
| GEE | Sigma-Aldrich | | Cat#G6503 | |
| IkB | Cell Signaling Technology | | Cat# 9242 | |
| Lactacystin | Enzo Life Sciences | | Cat# BML-PI104 | |
| MG132 | Sigma-Aldrich | | Cat# M8699 | |
| Myc-tag | Cell Signaling Technology | | Cat# 2278 | |
| NAC | Sigma-Aldrich | | Cat#A7250 | |
| NF-kB p65 | Santa Cruz Biotechnology | | Cat# sc-8008 | |
| NF-kB p65 (CHIP) | Santa Cruz Biotechnology | | Cat# sc-372X | |
| Nrf2 | Santa Cruz Biotechnology | | Cat# sc-13032 | |
| p62 | Santa Cruz Biotechnology | | Cat# sc-28359 | |
| Parkin | Cell Signaling Technology | | Cat# 4211 | |
| PARP | Cell Signaling Technology | | Cat# 9542 | |
| Pepstatin A | Enzo Life Sciences | | Cat# ALX-260-085 | |
| p-p65 (ser276) | Cell Signaling Technology | | Cat# 3037 | |
| Pro-caspase3 | Enzo Life Sciences | | Cat# ADI-AAP-113 | |
| PSMA5 | Cell Signaling Technology | | Cat# 2457 | |
| PSMB5 | Cell Signaling Technology | | Cat# 12919 | |
| Ref1 | Santa Cruz Biotechnology | | Cat# sc-5572 | |
| RNF183 | Invitrogen | | Cat# PA5-23862 | |
| Survivin | R&D Systems | | Cat# AF886 | |
| TNF-α | R&D Systems | | Cat# P01375 | |
| TRAIL | R&D Systems | | Cat# 375-TL | |
| Ub | Santa Cruz Biotechnology | | Cat# sc-8017 | |
| Ub-HRP | Enzo Life Sciences | | Cat# BML-PW0150-0100 | |
| XIAP | BD Biosciences | | Cat# 610762 | |
| z-VAD-fmk | R&D Systems | | Cat# FMK001 | |
| **siRNAs** |  | |  | |
| Cathepsin D siRNA | Santa Cruz Biotechnology | | Cat# sc-29239 | |
| GFP (control) siRNA | Bioneer | | 5`- GUU CAG CGU GUC CGG CGA G -3` | |
| NF-κB p65 siRNA | Santa Cruz Biotechnology | | Cat# sc-29410 | |
| Nrf2 siRNA | Santa Cruz Biotechnology | | Cat# sc-7049 | |
| p62 siRNA | Santa Cruz Biotechnology | | Cat# sc-29679 | |
| Parkin siRNA | Santa Cruz Biotechnology | | Cat# sc-42158 | |
| PSMA5 siRNA | Santa Cruz Biotechnology | | Cat# sc-62882 | |
| RNF183 siRNA | Bioneer | | 5`- CC CUC AGU UCC GCA UCU UU -3` | |
| **Plasmids** |  | |  | |
| Myc-RNF183 | Sino Biological | | Cat# HG16287-CM | |
| pRK5-HA-Ubiquitin | Addgene | | Cat# 17608 | |
| **Oligonucleotides** |  | |  | |
| H-actin-R | Bioneer | | 5`- CGA TTT CCC GCT CGG CCG TGG -3` | |
| H-Bcl-xL-F | Bioneer | | 5`- ATG GCA GCA GTA AAG CAA GCG C -3` | |
| H-Bcl-xL-R | Bioneer | | 5`- TTC TCC TGG TGG CAA TGG CG -3` | |
| H-p62-F | Bioneer | | 5`- AAG GCC TAC CTT CTG GGC AA -3` | |
| H-p62-R | Bioneer | | 5`- GCC ATC GCA GAT CAC ATT GG -3` | |
| H-PSMA5-F | Bioneer | | 5`- AGC AAT TGG CTC TGC TTC AG -3` | |
| H-PSMA5-R | Bioneer | | 5`- GCA TTC AGC TTC TCC TCC AT -3` | |
| H-PSMB5-F | Bioneer | | 5`- GTG AAG GGA ACC GGA TTT CAG -3` | |
| H-PSMB5-R | Bioneer | | 5`- CTC GAC GGG CCA GAT CAT AG -3` | |
| H-RNF183-F (qPCR) | Bioneer | | 5`- CGA AAA GCT TGA AGG ACT GG -3` | |
| H-RNF183-R (qPCR) | Bioneer | | 5`- TGA AGC AGC TCC AGT GAG AA -3` | |
