# Supplementary material for: Cathepsin D as a potential therapeutic target to enhance anticancer drug-induced apoptosis via RNF183-mediated destabilization of Bcl-xL in cancer cells
Source: Cell Death Dis. 2022 Feb 4;13(2):115. doi: 10.1038/s41419-022-04581-7 (PMC8816936; doi:10.1038/s41419-022-04581-7)
Supplement: Supplementary file 3 — co-authorship agreement [file 41419_2022_4581_MOESM3_ESM.pdf]

1. Seung Un Seo

Re: CDDIS-21-3207RRR Initial Quality Check

보낸 사람 서승언 <ssu3885@gmail.com> 받은 날짜 2022-01-22 12:47:52

받는 사람 권택규 중요도 보통

Hello, co-researcher

Recently, the Cell Death & Disease thesis (CDDIS-21-3207RRR) has been accepted, so I will send you an e-mail with the necessary documents for "Initial Quality Check" during the final file uploading process.

During the thesis revision process, HonhchanLee and Dr. Professor Hyun-Shik Lee provided a lot of experimental help and made me a co-author.

Regarding Honhchan Lee and Dr. Hyun-Shik Lee, the consent of all authors is required as co-authors.

Agree (O) Disagree ( )

Please make a decision as soon as possible and send me an email.

Sincerely yours

2. Seon Min Woo

Re: CDDIS-21-3207RRR Initial Quality Check

보낸 사람 우선민 <woosm724@gmail.com> 받은 날짜 2022-01-22 13:22:34

받는 사람 권택규 중요도 보통

Recently, the Cell Death & Disease thesis (CDDIS-21-3207RRR) has been accepted, so I will send you an e-mail with the necessary documents for "Initial Quality Check" during the final file uploading process.

During the thesis revision process, HonhchanLee and Dr. Professor Hyun-Shik Lee provided a lot of experimental help and made me a co-author.

Regarding Honhchan Lee and Dr. Hyun-Shik Lee, the consent of all authors is required as co-authors.

Agree (O) Disagree ( )

Please make a decision as soon as possible and send me an email.

Sincerely yours

3. Seung Soon Im

Re: CDDIS-21-3207RRR Initial Quality Check

보낸 사람 Seung Soon Im <ssoonim73@gmail.com> 받은 날짜 2022-01-22

받는 사람 권택규

Dear Prof. Taeq Kyu Kwon,

I agree to add Honchan Lee and Dr. Hyun-Shik Lee as co-authors.

Agree (o) Disagree (   )

Thanks

Seung-Soon Im

4. Younghoon Jang

[RE]전달: CDDIS-21-3207RRR Initial Quality Check

보낸 사람 장영훈 <yhjang@changwon.ac.kr> 받은 날짜 2022-01-22

받는 사람 권택규

Hi Professor Kwon,

I totally agree.

Regarding Honhchan Lee and Dr. Hyun-Shik Lee, the consent of all authors is required as co-authors.
Agree (o) Disagree ( )

Sincerely,

Younghoon Jang Ph.D.
Department of Biology and Chemistry
Changwon National University
Assistant Professor
Changwon, Gyeongnam, 51140, KOREA
TEL : +82 55 213 3458
FAX : +82 55 213 3459
C.P. : +8210 4640 8288
E-mail : yhjang@changwon.ac.kr

5. Eugene Han

회신: 전달: CDDIS-21-3207RRR Initial Quality Check

보낸 사람 한유진 <eghan@dsmc.or.kr> 받은 날짜 2022-01-22 16:52:13

받는 사람 권택규 중요도 보통

Dear Prof. Kwon

I agreed with your decision.

Thank you.

Eugene Han.

보낸 사람 : 권택규 <kwontk@kmu.ac.kr>
보낸 날짜 : 2022-01-22 12:28:27 (+09:00)
받는 사람 : "woosm724@gmail.com" <woosm724@gmail.com>,"ssu3885@gmail.com" <ssu3885@gmail.com>,"ssoonim73@gmail.com" <ssoonim73@gmail.com>,"yhjang@changwon.ac.kr" <yhjang@changwon.ac.kr>,"eghan@dsmc.or.kr" <eghan@dsmc.or.kr>,"shkim72@knu.ac.kr" <shkim72@knu.ac.kr>,"leehongchan@hanmail.net" <leehongchan@hanmail.net>,"leeh@knu.ac.kr" <leeh@knu.ac.kr>,"namjo@knu.ac.kr" <namjo@knu.ac.kr>,"egabriel@jhmi.edu" <egabriel@jhmi.edu>,"kjmin@dgmif.re.kr" <kjmin@dgmif.re.kr>,권택규 <kwontk@kmu.ac.kr>
참조 :
제목 : 전달: CDDIS-21-3207RRR Initial Quality Check

Hello, co-researcher

Recently, the Cell Death & Disease thesis (CDDIS-21-3207RRR) has been accepted, so I will send you an e-mail with the necessary documents for “Initial Quality Check” during the final file uploading process.

During the thesis revision process, HonhchanLee and Dr. Professor Hyun-Shik Lee provided a lot of experimental help and made me a co-author.

Regarding Honhchan Lee and Dr. Hyun-Shik Lee, the consent of all authors is required as co-authors.

Agree (O) Disagree ( )

Please make a decision as soon as possible and send me an email.

6. Sang Hyun Kim

[RE]전달: CDDIS-21-3207RRR Initial Quality Check

보낸 사람 김상현 | 받은 날짜 2022-01-22 17:18:30

받는 사람 권택규 | 중요도 보통

권택규

****
경북대학교 의과대학 약리학교실
대구광역시 중구 국채보상로 680, 신관455호
Tel: 053-420-4838
E-mail: shkim72@knu.ac.kr

Sang-Hyun Kim, Professor
Department of Pharmacology
School of Medicine
Kyungpook National University
680, Gukchaebosang-ro, Joong-gu
Daegu 41944, Reoublic of Korea
Tel: 82-53-420-4838
****

----- Original Message -----
From : 권택규 <kwontk@kmu.ac.kr>
To : "woosm724@gmail.com" <woosm724@gmail.com>, "ssu3885@gmail.com" <ssu3885@gmail.com>, "ssoonim73@gmail.com" <ssoonim73@gmail.com>, "yhjang@changwon.ac.kr" <yhjang@changwon.ac.kr>, "eghan@dsmc.or.kr" <eghan@dsmc.or.kr>, "shkim72@knu.ac.kr" <shkim72@knu.ac.kr>, "leehongchan@hanmail.net" <leehongchan@hanmail.net>, "leeh@knu.ac.kr" <leeh@knu.ac.kr>, "namjo@knu.ac.kr" <namjo@knu.ac.kr>, "egabriel@jhmi.edu" <egabriel@jhmi.edu>, "kjmin@dgmif.re.kr" <kjmin@dgmif.re.kr>, "권택규" <kwontk@kmu.ac.kr>
Cc :
Sent : 2022-01-22 12:28:31
Subject : 전달: CDDIS-21-3207RRR Initial Quality Check

Hello, co-researcher

Recently, the Cell Death & Disease thesis (CDDIS-21-3207RRR) has been accepted, so I will send you an e-mail with the necessary documents for “Initial Quality Check” during the final file uploading process.

During the thesis revision process, HonhchanLee and Dr. Professor Hyun-Shik Lee provided a lot of experimental help and made me a co-author.

Regarding Honhchan Lee and Dr. Hyun-Shik Lee, the consent of all authors is required as co-authors.

Agree (0) Disagree ( )

7. Hongchan Lee

RE: 전달: CDDIS-21-3207RRR Initial Quality Check

보낸 사람 이홍찬 <leehongchan@hanmail.net> 받은 날짜 2022-01-22 12:56:[illegible]

받는 사람 권택규 중요도 보[illegible]

이상입니다, 감사합니다.

이홍찬 올림.

--------- 원본 메일 ---------

> **보낸사람**: 권택규 <kwontk@kmu.ac.kr>
> **받는사람**: woosm724@gmail.com <woosm724@gmail.com>, ssu3885@gmail.com <ssu3885@gmail.com>, ssoonim73@gmail.com <ssoonim73@gmail.com>, yhjang@changwon.ac.kr <yhjang@changwon.ac.kr>, eghan@dsmc.or.kr <eghan@dsmc.or.kr>, shkim72@knu.ac.kr <shkim72@knu.ac.kr>, leehongchan@hanmail.net <leehongchan@hanmail.net>, leeh@knu.ac.kr <leeh@knu.ac.kr>, namjo@knu.ac.kr <namjo@knu.ac.kr>, egabriel@jhmi.edu <egabriel@jhmi.edu>, kjmin@dgmif.re.kr <kjmin@dgmif.re.kr>, 권택규 <kwontk@kmu.ac.kr>
> **날짜**: 22.01.22 12:28 GMT +0900
> **제목**: 전달: CDDIS-21-3207RRR Initial Quality Check
>
> Hello, co-researcher
>
> Recently, the Cell Death & Disease thesis (CDDIS-21-3207RRR) has been accepted, so I will send you an e-mail with the necessary documents for "Initial Quality Check" during the final file uploading process.
>
> During the thesis revision process, HonhchanLee and Dr. Professor Hyun-Shik Lee provided a lot of experimental help and made me a co-author.
>
> Regarding Honhchan Lee and Dr. Hyun-Shik Lee, the consent of all authors is required as co-authors.
>
> Agree (O) Disagree ( )
>
> Please make a decision as soon as possible and send me an email.
>
> Sincerely yours

8. Hyun-Shik Lee

[RE]전달: CDDIS-21-3207RRR Initial Quality Check

보낸 사람 이현식 <leeh@knu.ac.kr> 받은 날짜 2022-01-2[illegible]

받는 사람 권택규 중[illegible]

Dear Dr. Kwon,

I agree it.
Many thanks for your help.

Hyun-Shik

경북대학교
KYUNGPOOK NATIONAL UNIVERSITY

이현식

교수 | 자연과학대학 생명과학부
T 053-950-5300 | M 010-8980-1296 | E leeh@knu.ac.kr
A 대구광역시 북구 대학로80 자연과학대학 생명과학부

9. Ju-Ock Nam

[RE]전달: CDDIS-21-3207RRR Initial Quality Check

보낸 사람 남주옥 <namjo@knu.ac.kr> 받은 날짜 2022-01-22 13:49:10

받는 사람 권택규 중요도 보통

Dear Professor Kwon,

I agree.

Sincerely yours,

Ju-Ock Nam

10. Edward Gabrielson

전달: FW: CDDIS-21-3207RRR Initial Quality Check

보낸 사람 권택규 <kwontk@dsmc.or.kr> 받은 날짜 2022-01-24 09:28:

받는 사람 권택규 중요도 보

보낸 사람 : Edward Gabrielson <egabriel@jhmi.edu>
보낸 날짜 : 2022-01-22 21:09:12 (+09:00)
받는 사람 : 권택규 <kwontk@dsmc.or.kr>
참조 :
제목 : FW: CDDIS-21-3207RRR Initial Quality Check

Dr. Kwon,

I agree to Drs. HonhchanLee and Hyun-Shik Lee being co-authors.

Agree (X)   Disagree ( )

Edward Gabrielson

11. Kyoung jin Min

RE: 전달: CDDIS-21-3207RRR Initial Quality Check

보낸 사람 "민경진" <kjmin@kmedihub.re.kr> 받은 날짜 2022-01-22 12:48:52

받는 사람 권택규 중요도 보통

———— **원본 메일** ————

보낸사람 : "권택규" <kwontk@kmu.ac.kr>
받는사람 : "woosm724@gmail.com" <woosm724@gmail.com>, "ssu3885@gmail.com" <ssu3885@gmail.com>, "ssoonim73@gmail.com" <ssoonim73@gmail.com>, "yhjang@changwon.ac.kr" <yhjang@changwon.ac.kr>, "eghan@dsmc.or.kr" <eghan@dsmc.or.kr>, "shkim72@knu.ac.kr" <shkim72@knu.ac.kr>, "leehongchan@hanmail.net" <leehongchan@hanmail.net>, "leeh@knu.ac.kr" <leeh@knu.ac.kr>, "namjo@knu.ac.kr" <namjo@knu.ac.kr>, "egabriel@jhmi.edu" <egabriel@jhmi.edu>, "kjmin@dgmif.re.kr" <kjmin@dgmif.re.kr>, "권택규" <kwontk@kmu.ac.kr>
받은날짜 : 2022-01-22 (토) 12:28:36
제목 : 전달: CDDIS-21-3207RRR Initial Quality Check

Hello, co-researcher

Recently, the Cell Death & Disease thesis (CDDIS-21-3207RRR) has been accepted, so I will send you an e-mail with the necessary documents for “Initial Quality Check” during the final file uploading process.

During the thesis revision process, HonhchanLee and Dr. Professor Hyun-Shik Lee provided a lot of experimental help and made me a co-author.

Regarding Honhchan Lee and Dr. Hyun-Shik Lee, the consent of all authors is required as co-authors.

Agree (O)   Disagree ( )

Please make a decision as soon as possible and send me an email.

Sincerely yours

12. Taeg Kyu Kwon

전달: CDDIS-21-3207RRR Initial Quality Check

보낸 사람 권택규 <kwontk@kmu.ac.kr> 받은 날짜 2022-01-24 09:13:3

받는 사람 권택규 중요도 보통

보낸 사람 : "권택규" <kwontk@kmu.ac.kr>
보낸 날짜 : 2022-01-22 12:28:23 (09:00)
받는 사람 : woosm724@gmail.com <woosm724@gmail.com>, ssu3885@gmail.com <ssu3885@gmail.com>, ssoonim73@gmail.com <ssoonim73@gmail.com>, yhjang@changwon.ac.kr <yhjang@changwon.ac.kr>, eghan@dsmc.or.kr <eghan@dsmc.or.kr>, shkim72@knu.ac.kr <shkim72@knu.ac.kr>, leehongchan@hanmail.net <leehongchan@hanmail.net>, leeh@knu.ac.kr <leeh@knu.ac.kr>, namjo@knu.ac.kr <namjo@knu.ac.kr>, egabriel@jhmi.edu <egabriel@jhmi.edu>, kjmin@dgmif.re.kr <kjmin@dgmif.re.kr>, 권택규 <kwontk@kmu.ac.kr>
참조 :
제목 : 전달: CDDIS-21-3207RRR Initial Quality Check

Hello, co-researcher

Recently, the Cell Death & Disease thesis (CDDIS-21-3207RRR) has been accepted, so I will send you an e-mail with the necessary documents for "Initial Quality Check" during the final file uploading process.

During the thesis revision process, HonhchanLee and Dr. Professor Hyun-Shik Lee provided a lot of experimental help and made me a co-author.

Regarding Honhchan Lee and Dr. Hyun-Shik Lee, the consent of all authors is required as co-authors.

Agree (0) Disagree ( )

Please make a decision as soon as possible and send me an email.

Sincerely yours
